# Treatment of a Chronic Vulvar Wound by Hyperbaric Oxygen Therapy (HBOT)

**DOI:** 10.1155/2022/6083915

**Published:** 2022-01-11

**Authors:** D. Ricard-Gauthier, M.-A. Panchard, D. E. Huber

**Affiliations:** ^1^Department of Gynecology and Obstetrics, Valais Hospital, Sion, Switzerland; ^2^Department of Underwater and Hyperbare, University Hospital of Geneva, Switzerland

## Abstract

We hereby report the case of a 66-year-old obese patient (BMI 30) with type 2 diabetes, who presented a chronic vulvar lesion on the left labia majora following surgical drainage of an abscess. After multiple unsuccessful treatments by antibiotics and local wound care, we proposed a trial of hyperbaric oxygen therapy (HBOT). The patient fully recovered after 54 sessions at 2.5 ATA, 95 minutes each. HBOT has been studied for perineal lesion such as skin atrophy or necrosis caused by irradiation but not for vulvar nonhealing chronic lesions in the case of impaired vascularization caused by diabetes. This case is, to our knowledge, one of the first publications about the healing boost of HBOT in chronic vulvar wounds due to vascular impairment.

## 1. Background

Impaired healing causing chronic wounds is a constant and increasing challenge for the medical society with an estimation of 6.5 million cases per year [1]. Patient's quality of life is particularly compromised by this affliction which demands a lot of care and long-term treatments. During the past years, numerous techniques have been developed such as skin substitutes, wound dressing, injection of growth factors, negative pressure wound therapy, and hyperbaric oxygen therapy (HBOT) [1, 2]. HBOT is an established treatment for chronic nonhealing wounds of different origins: due to arterial insufficiency and mucosal ulcers postradiotherapy [3]. The treatment consists in breathing 100% oxygen in ambient pressure greater than 1 atmosphere absolute (ATA) in a chamber. At 2.5 ATA 100% O_2_, the total content is 24.2 ml O_2_/100 ml blood instead of 19.2 ml O_2_/100 ml at 1 ATA room air. Consequently, it saturates the blood with oxygen causing hyperoxia of the lesions and massively increases the blood oxygen content. The mechanisms of action for improving wound healing are numerous and varied: fibroblast proliferation, induction of collagen synthesis, neoangiogenesis, and antimicrobial defense [4]. Most of the studies focused their research on chronic wounds occurring after an oncological surgery or radiation and only a few on lesions caused by other origins [5–8].

In this case report, we describe the successful healing of a vulvar chronic wound using HBOT after numerous treatment failures.

## 2. Case Presentation

A 66-year-old obese patient (BMI 30) was referred to our department for vulvar pain and redness. The patient's medical history revealed type 2 diabetes treated by insulin aspart 30 U, insulin degludec 60 U, liraglutide 1-8 mg, hypertension treated by metoprolol 100 mg, cilazapril 5 mg, and finally atrial fibrillation with intake of rivaroxaban 20 mg. In 1992, she underwent a hysterectomy with bilateral salpingectomy. In March 2020, a perianal abscess, far from the vulva, was treated surgically with good skin healing. The patient had no arterial insufficiency or diabetic vasculopathy history.

In July 2020, the patient consulted in our department due to an increasing and painful swelling which appeared within two weeks. At examination, we observed a unilateral swelling with redness of the left labia majora. We diagnosed a vulvar abscess of 5 × 3 cm and proposed a conservative treatment with local application of Ichtyol. After two days of treatment, no improvement was observed and surgical treatment of the abscess by incision with drainage was decided. We made a 2 cm incision on a 1 cm depth and 5 cm length collection. Local swab of a brownish and odorous liquid considered as pus remained negative for any pathogen. Biopsies showed inflammatory change but no malignant signs.

**Figure 1:**
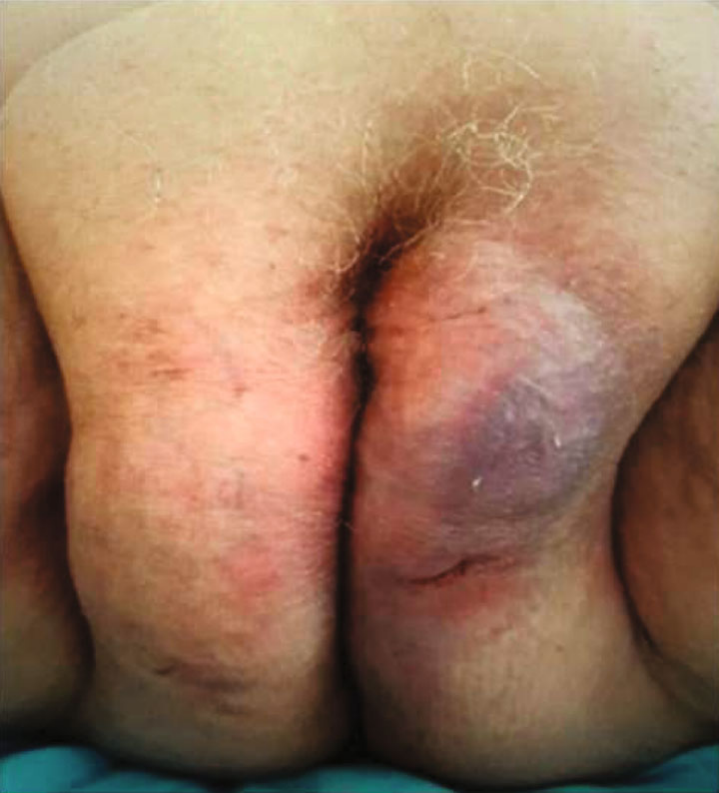
Incision site of the labia majora with an impaired healing and suspicion of cellulitis.

After surgery, we observed impaired wound healing with the development of severe skin swelling and redness of the labia (Figure 1). We suspected a cellulitis and decided to treat the patient with clindamycin 600 mg per os two times per day for seven days. No improvement was noted, and new swabs showed the presence of *E. faecalis*. We decided to introduce a treatment of cotrimoxazole two times per day for 10 days. Even after this second treatment, there was still no healing, and as a result, the patient was hospitalized for IV treatment with vancomycin 1.5 g two times per day and ciprofloxacin 500 mg two times per day for 18 days.

**Figure 2:**
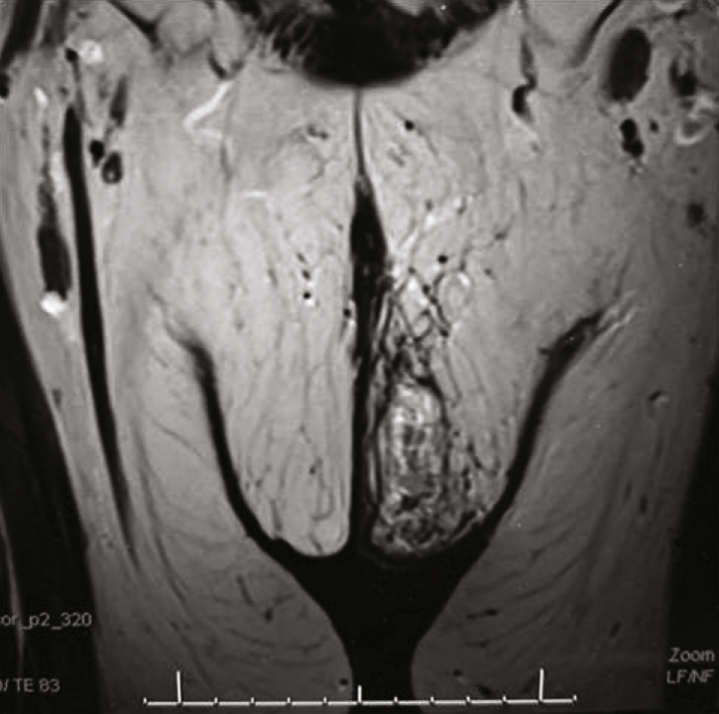
MRI showing an empty cavity without any collection. Skin infiltration with suspicion of a cellulitis. Absence of fistula.

MRI confirmed vulvar cellulitis as well as the remnants of a 2.8 cm over 2.1 cm cavity without any new abscess or fistula (Figure 2). A swab of the lesion was negative for bacteria following antibiotic therapy. Following these multiple unsuccessful treatments during 4 weeks, we proposed HBOT. The patient did not present any contraindications for this therapy.

**Figure 3:**
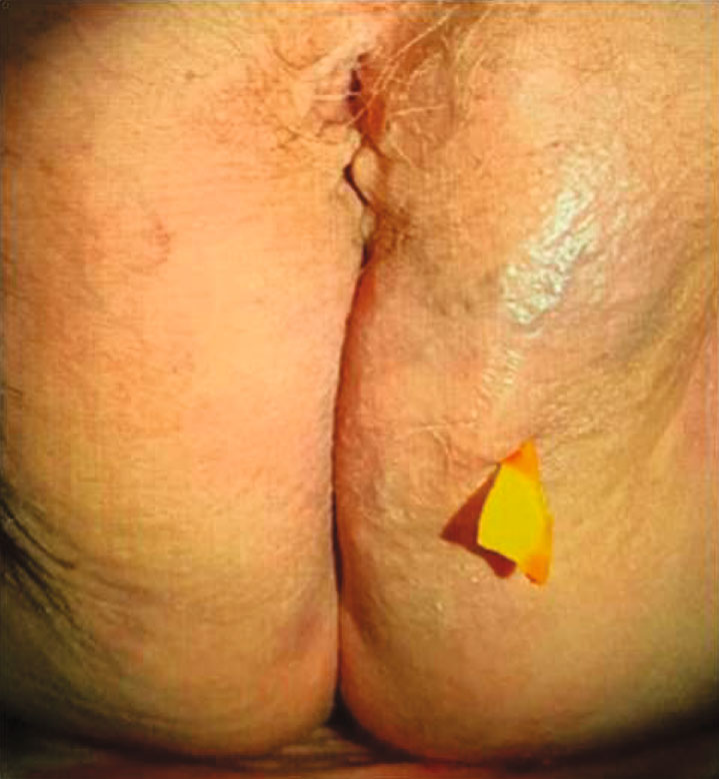
Image of the labia majora after the first session of HBOT.

**Figure 4:**
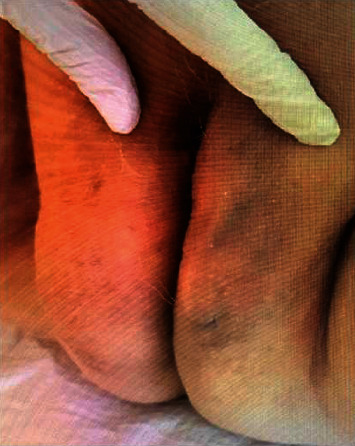
Image of the labia majora at the end of the treatment with complete healing of the incision site.

For the HBOT, the patient was hospitalized in the Department of Underwater and Hypercare Medicine, at the University Hospital of Geneva. Two sessions at 2.5 ATA, 95 minutes each, were done from Monday to Friday and one session every Saturday. Local treatment of the lesion was done with disinfection by aqueous chlorhexidine once a day. After 54 sessions, the patient presented full recovery with only a small skin lesion with no remaining cavity (Figures 3 and 4).

## 3. Discussion

For vulvar chronic wounds, negative pressure therapy, application of platelet gel, and growth factors have been used with good healing results [2, 9]. However, when lesions are small with cutaneous fistula, these options can be laborious with limited access. As such, HBOT could be a new option for those specific cases. Moreover, healing requires oxygen in order to be used by the immune system and new proliferating cells [1]. Wounds with low oxygen saturation are more at risk of necrosis and impaired healing [10]. In our case, the patient suffered from diabetes causing impaired wound healing due to a multifactorial origin (vascular, neuropathic, immune, and biochemical disturbance). In consequence, with difficult access of the wound and probable low oxygen saturation in the context of diabetes, we think this case is perfect for HBOT. This treatment has minimal side effects, is unpainful, and is easy to apply. There are only few absolute contraindications such as untreated pneumothorax, decompensated pulmonary obstructive disease, or decompensated cardiac failure [11]. On the other hand, the treatment can be long with the need of repeated sessions requiring organization. In our case, we were able to observe, full recovery after HBOT and minimal local treatment. The patient complained about the time needed for this method with long-lasting hospitalization; however, she was very satisfied with the final result.

In our knowledge, this case is the first to report an efficient treatment by HBOT of a nonhealing chronic lesion not caused by irradiation. This supports HBOT as an optional treatment for impaired vulvar wound healing. As such, we believe that more studies should concentrate on the use of HBOT for the healing of chronic vulvar lesions.
